# Confounding in Epidemiological Studies on Assessment of the Impact of Genetic Factors on Disease Risk: The Problem of Redundant Adjustment

**DOI:** 10.2188/jea.JE20230277

**Published:** 2024-10-05

**Authors:** Yumiko Kasugai, Isao Oze, Yuriko N. Koyanagi, Yukari Taniyama, Hidemi Ito, Issei Imoto, Keitaro Matsuo

**Affiliations:** 1 Division of Cancer Epidemiology and Prevention, Aichi Cancer Center Research Institute, Nagoya, Japan; 2 Division of Cancer Information and Control, Aichi Cancer Center Research Institute, Nagoya, Japan; 3 Department of Descriptive Cancer Epidemiology, Nagoya University Graduate School of Medicine, Nagoya, Japan; 4 Aichi Cancer Center Research Institute, Nagoya, Japan; 5 Department of Cancer Epidemiology, Nagoya University Graduate School of Medicine, Nagoya, Japan

In epidemiological studies focusing on gene variants as the exposure of interest, the selection of adjustment variables is often done conventionally. Most studies typically choose age and sex as adjustment variables, and often additionally include factors like smoking and alcohol consumption, which are risk factors for the disease, for further adjustment.

There are three traditional criteria of a confounder: a) it must be associated with the exposure; b) it must be associated with outcome among the unexposed; and c) it must not lie on a causal pathway between exposure and outcome.^1^ However, these criteria serve only as necessary conditions. Using directed acyclic graphs (DAGs), confounding is defined by the presence of variables that intercept confounding paths between exposure and outcome.^2^ Furthermore, practical methods for the selection of confounders, such as the Disjunctive Cause Criterion, have been proposed.^3^

The selection of confounders requires knowledge essential for estimating causal inference. Previous Genome-Wide Association Studies (GWAS) have revealed that gene variants are associated with various diseases and lifestyle factors. As often seen in Mendelian Randomization studies, where the assumption of horizontal pleiotropy may not hold, some gene variants are associated with multiple phenotypes.^4^ Therefore, conventional selection of covariates might lead to inappropriate adjustment, such as adjustment for intermediate factors.

In this letter, we would like to reconsider confounding factors for epidemiologic studies that assess the contribution of gene variants to disease as exposures in terms of causal inference. In our previous article,^5^ using data from the Hospital-based Epidemiological Research Program at Aichi Cancer Center (HERPACC), we assessed the impact of Germline Pathogenic Variants (GPVs) of cancer-predisposing genes, such as *BRCA1* or *BRCA2*, on breast cancer risk. We found that GPVs did not confound with environmental factors or with single nucleotide polymorphisms (SNPs) identified in GWAS.^6^ To our knowledge, this study was the first to demonstrate that GPVs do not confound with environmental factors in assessing breast cancer risk. In this letter, we aim to show that this is also the case with SNPs as exposures. Further, we propose minimum requirements in epidemiologic studies aimed at evaluating the association between exposure to genetic factors such as SNPs, GPVs, and other genetic variants and to disease risk.

**Figure 1.**
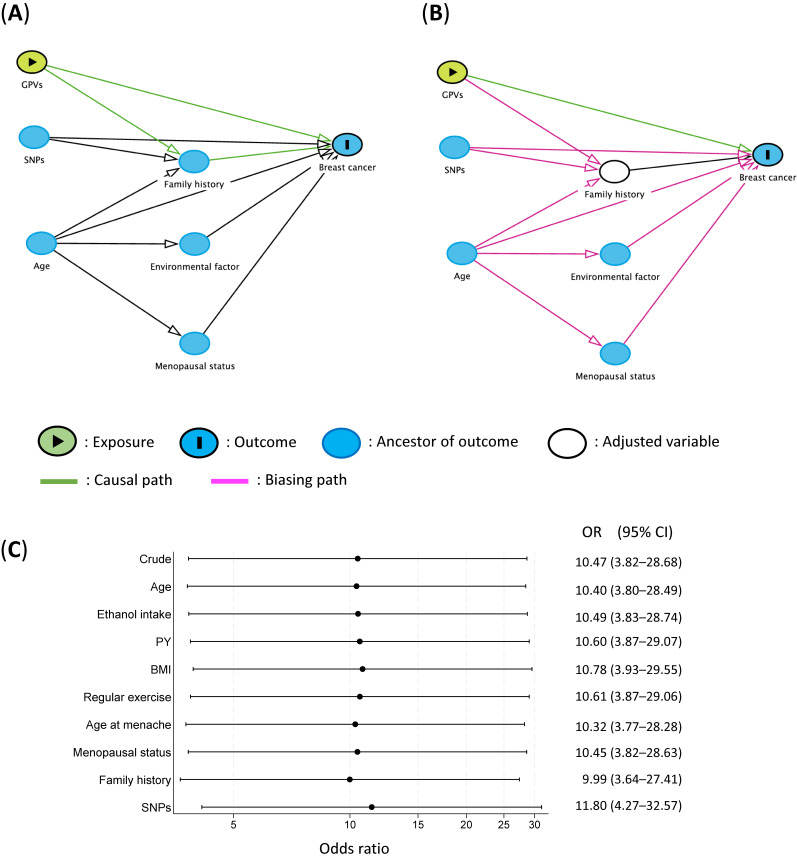
(**A**) and (**B**) Directed acyclic graphs for causal estimation of breast cancer incidence created in DAGitty. (**A**) Minimal adjustment sets containing age, environmental factors, and menopausal status for estimating the total effect of GPVs on breast cancer. No adjustment is necessary to estimate the total effect of GPVs on breast cancer. (**B**) The total effect cannot be estimated due to adjustment for an intermediate. (**C**) Environmental factors influencing the association between GPVs and breast cancer risk overall. BMI, body mass index; GPVs, germline pathogenic variants; OR, odds ratio; PY, pack year; SNPs, single nucleotide polymorphisms.

Using a breast cancer case-control dataset (625 cases and 1,133 controls) used in a previous study,^5^ we used directed acyclic graphs (DAGs) to visualize a model to assess the impact of GPVs or SNPs on breast cancer in individual samples.^5^^,^^7^ Details of the case-control study have been described elsewhere.^5^ Figure 1A shows a minimal set of adjustments, including age, SNPs, environmental factors, and menopausal status, required to estimate the overall impact of GPVs on breast cancer. When observing the association of breast cancer with GPVs as an exposure, SNPs and age are not upstream of GPVs and both are independent risk factors. Because family members from a genetic background with GPVs have a higher incidence of cancer than those without, family history can be considered an intermediate factor between GPVs and breast cancer. Given this, we excluded family history from the multivariable model. Inclusion would have prevented an unbiased evaluation of the causal relationship between GPVs and breast cancer (Figure 1B). When evaluating GPVs as exposure, there is no need to adjust for any other risk factors because they are not upstream of GPVs and are not confounded by them.^3^

To validate this perspective, we performed sensitivity analyses, individually adding each variable (age, alcohol intake, and pack years [PY] of smoking, body mass index [BMI], exercise habits, age at menarche, menopausal status, family history of breast cancer, and SNPs) to the crude analysis. We confirmed that, as designated in the DAG (Figure 1C), the GPVs were not appreciably confounded with any of these factors, except for SNPs. A degree of correlation between GPVs and SNPs may be present, indicating potential confounding. GPVs and SNPs are located apart on the chromosome and are independent factors. Therefore, a correlation between GPVs and SNPs is unlikely. It is considered that the change in ORs occurred by chance. Adjusting for family history also adds bias but did not have much effect on the actual results of the analysis. While the DAG can visually depict the causal relationship among various factors, it does not illustrate their magnitude.

**Figure 2.**
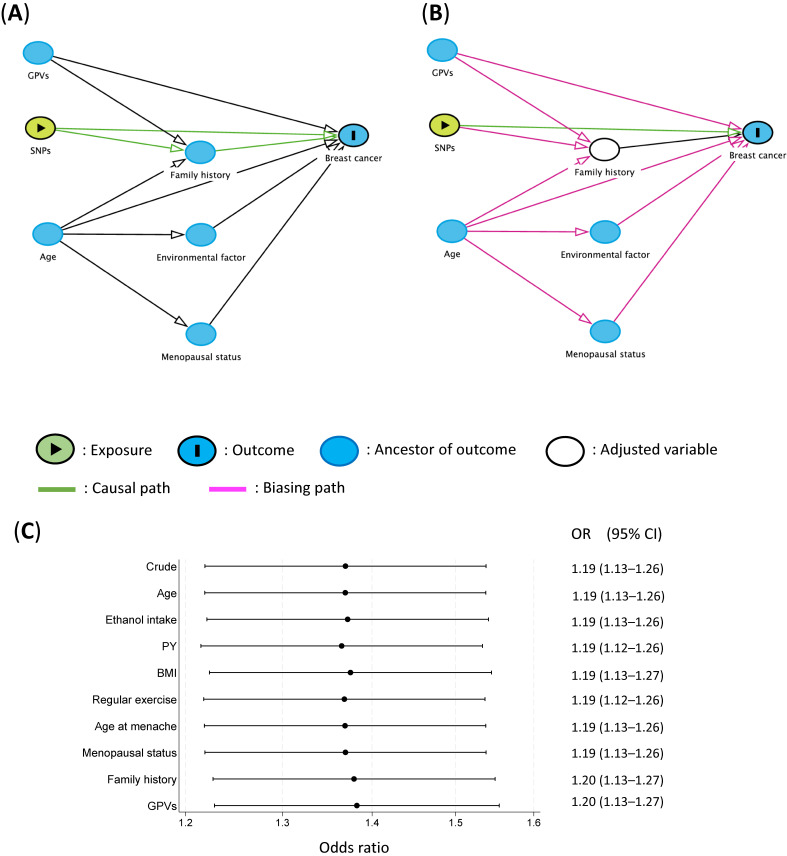
(**A**) and (**B**) Directed acyclic graphs for causal estimation of breast cancer incidence created in DAGitty. (**A**) Minimal adjustment sets containing age, SNPs, environmental factors, and menopausal status for estimating the total effect of SNPs on breast cancer. No adjustment is necessary to estimate the total effect of SNPs on breast cancer. (**B**) The total effect cannot be estimated due to adjustment for an intermediate. (**C**) Environmental factors influencing the association between SNPs and breast cancer risk overall. BMI, body mass index; GPVs, germline pathogenic variants; OR, odds ratio; PY, pack year; SNPs, single nucleotide polymorphisms.

Similarly, when evaluating the association between SNPs and breast cancer risk, we used these DAGs to identify factors that required adjustment and then confirmed these through sensitivity analysis. Both GPVs and age were independent of SNPs (Figure 2A), and a family history of breast cancer served as an intermediate factor (Figure 2B). Any environmental factors directly influenced by SNPs must be excluded from adjustment, as they also serve as intermediate factors. These were indeed excluded from this study. When setting SNPs as exposure in this example, there is theoretically no need to adjust for any other risk factors, as they do not confound with each other.^3^ Supporting this notion, and similar to the previous example concerning GPVs and breast cancer risk, none of the variables affected the ORs for the effects of SNPs on breast cancer (Figure 2C).

Matching on confounders in case-control studies can introduce a collider bias in the ‘exposure → confounder → selection ← outcome’ pathway. However, as gene variants do not have confounders, matching in our study does not result in collider bias.^8^

In this study, we conducted an epidemiological evaluation of the association between germline genetic variants, namely GPVs and SNPs, and disease risk using a breast cancer case-control study as an example. We used DAGs to organize other established breast cancer risk factors and performed a sensitivity analysis to confirm whether any confounding existed in the association.

**Figure 3.**
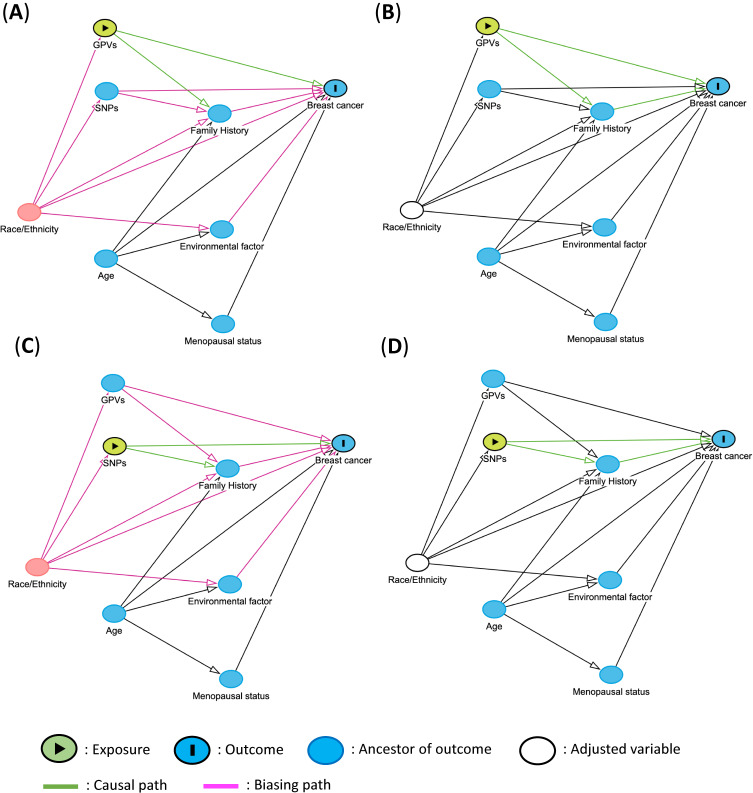
Directed acyclic graphs for causal estimation of breast cancer incidence created in DAGitty. (**A**) Minimal sufficient adjustment sets containing Race/Ethnicity for estimating the total effect of GPVs on breast cancer. (**B**) After adjusting for Race/Ethnicity, the bias pathway closed. (**C**) Minimal sufficient adjustment sets containing Race/Ethnicity for estimating the total effect of SNPs on breast cancer. (**D**) After adjusting for Race/Ethnicity, the bias pathway closed. GPVs, germline pathogenic variants; SNPs, single nucleotide polymorphisms.

In the example of the case-control study conducted within a single population, adjustment for population is not required. However, in studies involving multiple populations, population adjustment is essential, as a population can influence both the distribution of gene variants and the frequency of diseases (Figure 3).^9^ Most other variables do not affect genetic variants, so their adjustment does not introduce bias. However, intermediate factors that are associated with genetic variants can influence the outcome and may introduce bias through adjustment. If it is difficult to determine whether a factor is an intermediate factor, it might be helpful to present both crude and adjusted models.

This study suggests that, when examining the association between germline genetic variation and disease risk, no variables except for population need to be adjusted in multivariable models.
